# Environmental adaptability, morphometric features with reproductive and productive potentialities of indigenous sheep in Bangladesh

**DOI:** 10.5455/javar.2022.i634

**Published:** 2022-12-31

**Authors:** Md. Rezaul Hai Rakib, Nure Hasni Desha, Md. Zillur Rahman, Md. Ahsanul Kabir, Farzana Yasmin, Md. Ashadul Alam, Sonia Akther, Nasrin Sultana

**Affiliations:** 1 Training, Planning & Technology Testing Division, Bangladesh Livestock Research Institute, Dhaka, Bangladesh; 2 College of Veterinary Medicine, China Agricultural University, Beijing, China; 3 Sheep Production Research Division, Bangladesh Livestock Research Institute, Dhaka, Bangladesh; 4 Biotechnology Division, Bangladesh Livestock Research Institute, Dhaka, Bangladesh; 5 College of Animal Science and Veterinary Medicine, Huazhong Agricultural University, Wuhan, China; 6 Animal Production Research Division, Bangladesh Livestock Research Institute, Dhaka, Bangladesh; † These two authors contributed equally

**Keywords:** Biodiversity, diseases, farming, heat stress, nutrition, sheep

## Abstract

Indigenous sheep are highly adaptable and widely distributed in different regions of Bangladesh. They are famous for their tolerance to harsh environmental conditions, low demand for feed, and disease resistance with minimum or no housing and management facilities. Ample indiscriminate research reports and case studies on the native sheep of Bangladesh have been published. Nonetheless, a comprehensive review of reproductive and productive performances, as well as their various morphometric physiognomies and climate resilience capabilities, is lacking. This review was designed to explore and summarize the available research reports on indigenous sheep to highlight the gaps and provide an updated database for the future research plan for sustainable native sheep production in Bangladesh. It covers studies on sheep biodiversity, their adaptability to the local environment, morphometric features, feeding and nutrition, reproduction and production performances, diseases and health management, and the problems and prospects of sheep farming in Bangladesh. Due to the increased demand for animal protein, especially red meat, the scope of sheep farming increases along with that of other large and small ruminants. The vital constraints of sheep production in Bangladesh include insufficient feed and fodder supply with its high prices, higher disease occurrence with low or no management practices, kid mortality, and a poor marketing channel with the lower popularity of sheep meat than goat meat. Future research would be a prerequisite to measuring the impact of native sheep on household economies and food security during the year, evaluating the other challenges, and finding out the possible interventions in the fields of nutrition, reproduction, and health management.

## Introduction

Livestock plays a dynamic role in promoting the national economy of Bangladesh as it is one of the most important sub-sectors of agriculture [1]. In the rural areas of Bangladesh, about 80% to 85% of the households that are landless, marginal, or small farmers keep livestock to support their household income [2]. Among the livestock species, the small ruminant is a major component that contributes diversified to this sector [3]. In the national economy, small ruminant farming may make a huge contribution by generating income and creating job opportunities.

Sheep are Bangladesh’s third largest livestock population and one of the most important providers of total meat supply; there are approximately 3.607 million sheep in Bangladesh, and this population has increased by approximately 20% over the last decade [4,5]. However, Asia and Africa are the central hub (64.43%) of sheep populations worldwide, while about 25% of the total population and 233 of about 920 breeds are found in Asia [6]. The distinct features of sheep rearing include its higher production potential with smaller feed requirements, lower disease risks, and broader adaptability to different climatic conditions. In addition to the good meat, milk, fiber, and skin that they provide, they also make sure that food is safe and that households are stable.

In Bangladesh, most of the sheep are native non-descript types with a small number of exotics and crossbreeds, and here people rear sheep as their family practice [7]. The indigenous sheep (*Ovis aries*) of Bangladesh are descended from Asia’s wild Ural (*Ovis orientalis vignei*), which is mostly found in three ecological zones: Barind, the Jamuna river basin, and coastal areas [8]. Moreover, farmers are also found to rear some exotics and crossbreds like Garole and Muzaffarbadi crosses, which are very popular among the livestock farmers of the southern part (Sundarban delta region) and western border areas (Meherpur, Chuadanga, and Chapainawabganj districts, respectively) [9]. However, indigenous sheep are the most adaptable, extensively disseminated, and noticeable in Bangladesh. They are well known for their little demand for feed, tolerance to harsh climate conditions, and disease resistance with remarkably good-quality red meat and skin. It has also had a significant impact on poverty alleviation, women’s and young people’s employment and empowerment, and food security by improving the livelihoods of poor and marginal farmers. Therefore, it is often considered the “bank of the poor man” in some areas of Bangladesh. But the information related to native sheep production provided by the farmers in Bangladesh is very inadequate. Furthermore, in small-scale farming, little or no attention has been paid to promoting lamb or mutton for consumers. Lack of public consciousness, misunderstanding about sheep meat (lamb or mutton), and insufficient nutrition supplementation are the limiting factors for sheep farming in Bangladesh. Recently, the Bangladesh government has emphasized sheep farming to meet future demand for animal protein due to population growth. This study was commenced to review, discuss, summarize, and compare the existing literature to determine the phenotypic characteristics, important morphological traits, and reproductive and productive performances of indigenous sheep, as well as their prospects and problems in Bangladesh.

## Sheep Biodiversity in Bangladesh

**Figure 1.**
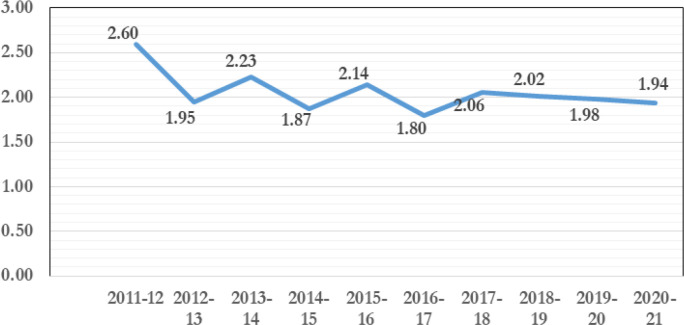
Growth rate (%) of sheep in last 10 years (Source: Department of Livestock Services, 2021).

In Bangladesh, most sheep population is mainly of the non-descriptive indigenous type, with some crossbreds [7,10]. The total sheep population of the country is about 3.68 million [11], with no remarkable changes in their population growth rate in the last 10 years, as shown in Figure 1. According to concentration, morphology, production, and reproduction performance, the indigenous sheep are categorized into three distinct types, *viz.* Coastal, Jamuna River Basin, and Barind [12,13], along with indigenous sheep, another common breed is Garole, which is found in the neighboring districts of Bangladesh’s Sundarban delta area and the Indian state of West Bengal [15,16] (Table 1). Phylogenetic analysis using mtDeoxyribonucleic Acid (mtDNA) sequence information found *O. aries* as the single ancestor of Bangladeshi indigenous sheep [17]. There is a high genetic dissimilarity within populations and a low genetic dissimilarity among sheep populations disseminated regionally [17]. Based on microsatellite marker evaluation, Barind and Jamuna river basin sheep belong to a related genetic group, while coastal sheep belong to other genetic groups [14]. However, based on mitochondrial DNA sequence, all Bangladeshi sheep populations are in the same group, and there is an evolutionary relationship with Indian, Chinese, and Turkish sheep breeds [17].

**Table 1.** Sheep biodiversity and distribution in Bangladesh.

Genotype	Breed/type	Distribution	References
Indigenous/native	Coastal	Coastal regions (Noakhali, Charlands, and other coastal plain area)	
	Jamuna river basin	Jamuna river basin areas (Tangail, Sirajganj, Gaibandha, Sherpur, Jamalpur, Mymensingh, and Dhaka specially, both sides of Jamuna river in Bangladesh)	[4,12,13,21,22]
	Barind	Barind tracts (Naogaon, Rajshahi, Chapai Nawabganj)	
	Garole	Sundarban delta region, southern part of Bangladesh	[9,16,23,24]	
Exotic	Suffolk		
	Perendale	BLRI, Savar, Dhaka	[19]
	Dorper		
	Muzaffarbadi	Western border areas (Meherpur, Choadanga and Chapainawabganj)	[9,20,25]
	Nagpuri/Chotanagpuri (Locally known as Garole in some areas)	Meherpur and Chapainawabganj district and the Sundarban delta region, southern part of Bangladesh	[9,14]
Crossbred	Muzaffarbadi cross	Western border areas (Meherpur, Choadanga, and Chapainawabganj districts)	[9,20,25]
	Nagpuri/Chotanagpuri cross	Meherpur and Chapainawabganj district and its adjacent regions	[14]

Several attempts had been made to improve the indigenous sheep germplasm with high-performing exotic sheep. In 1965, the Lohi breed of Pakistan; in 1976, the Romney Marsh breed of New Zealand; and in 1984, the Suffolk and Perendale breeds of Australia were imported to improve the indigenous sheep of coastal areas through crossbreeding, but the programs did not sustain themselves for a longer period [9,13–18]. Hence, the impact of the exotic sheep germplasm on the indigenous sheep is inconsiderable. In 2016, Bangladesh Livestock Research Institute (BLRI) imported Suffolk, Perendale, and Dorper from Australia to adapt the exotic germplasm and produce crossbreds to improve the production performances of indigenous sheep [19].

Nowadays, many exotic and crossbred sheep are entering Bangladesh from neighboring countries through illegal transportation, resulting in the availability of exotic sheep breeds and their crossbreds in the border areas. Muzaffarabad and Nagpuri sheep breeds, as well as crossbreds, have been discovered in Meherpur, Chuadanga, Chapainawabganj, and other border districts [10,14–20], disrupting the planned breeding system and causing genetic erosion of our valuable native sheep germplasm.

## Adapting to the Local Environment and Climate Change

Currently, the economic viability of the sheep production system is threatened due to the devastating effects of climate change because sheep are susceptible to the adverse impacts of climate change. Heat stress, cyclones, droughts, heavy rainfall, and unpredictable climates are the important factors that affect sheep husbandry, growth, and productivity [26]. Moreover, climate change leads to several vector-borne diseases and causes immune suppression in sheep [27,28]. But in extreme environmental conditions, sheep perform better under heat stress than other ruminants [29,30]. Usually, the more specialized and carefully chosen breeds are the ones that can handle being crowded the best and don’t react as much to predators or other things that make them scared.

Domestic sheep breed differences are discovered due to selection for breed traits and adaptation to various criteria, such as environmental adaptation, seasonality responses, and ability to cope with food scarcity [31]. Sheep’s behavioral, morphological, physiological, and largely genetic bases enable them to be highly adaptable [32] in both high mountains of hypoxia and extreme lowlands of a thermally stressed environment [33–35]. As with rodents, the integrated energy metabolisms of different sheep breeds are responsible for adaptation to extreme climates [36]. Sunlight, precipitation, and temperature—these three climatic variables—have effects directly on the thermoregulation of sheep and indirectly on metabolic regulation via depressing fodder quality and biomass yield [37]. Extreme sunlight plays a vital role in physiological events in sheep, like reproduction, and serves as a primary indication for the timing of reproduction events in sheep [38,39]. Increasing respiratory rate, panting, increasing sweating rate, changes in endocrine function, and reducing metabolic rate are the thermoregulation mechanisms used by sheep to maintain their body temperature in heat-stressed environments [40,41].

Morphological characteristics like body size and shape influence the thermoregulatory mechanisms of farm animals under heat stress [42]. Berihulay et al. [35] and Mwacharo et al. [43] reported that taller animals dissipate more heat with lower metabolic rates than animals with short, squat bodies, who gain heat at a slower rate. Besides, dark-colored sheep are more susceptible to heat stress because they absorb more heat than those with light pigmentation [44]. However, sheep with carpet-type wool, light-colored fleece, thinner skin, shorter hairs, and fatter tails are found to be better heat dissipators in hot weather [45]. About 25% of the world’s sheep population comprises fat-tailed breeds, and in extreme environments, it helps in the adaptive response of animals. It is a valuable energy reservoir for them in migration and the winter season [46].

Indigenous sheep breeds outperform exotic and crossbred sheep in terms of adaptability to the agro-climatic zones where they have evolved. Although the production performances of indigenous sheep breeds are lower than those of exotic and crossbred sheep, they keep a stable production by utilizing available poor-quality roughages with minimum concentrate supplementations under stressful climatic conditions, where high-producing sheep fail to maintain their minimum production performances [47].

## Morphometric Features

### Physical appearances

Physical appearances or features are the most commonly used breed or strain standards, and the main category of animal phenotypic expression results from both genotype and environment [12]. The coat color of indigenous sheep is mainly cream or white, but brown, white brown, dark brown, black-brown, gray, and gray with black or white patches are also found [12,13,48,49]. The face, ears, and feet are primarily light black. Males have horns, but females are polled. Ears are of small, medium, and long types. The tail is short and thin, but the wools are coarse with high medullation [13,48]. There is no such significant variation in coat color between areas. Still, in the case of ears, significantly shorter ears are found in Barind than in the Jamuna river basin and coastal areas [13]. Pervage et al. [12] found whitish, brown, blackish-red, and black-and-white mixed skin colors of sheep in the Jamuna River Basin and Barind region, but in the coastal area, mostly white or light to deep brown was available. In terms of body measurements, males outnumber females [50] (Table 2), and sheep from the coastal region have larger body measurements than those from the Barind and Jamuna River Basin regions [12,13,49]. However, differences between physical appearances might be due to hereditary and non-hereditary factors, such as breed, season, birth type, birth weight, and management [10].

### Lamb birth weight

Lamb birth weight is an early measurable growth trait positively correlated with further growth, organ development, and satisfactory production [51]. In native lamb, it ranges from 1.04 to 2.3 kg [12,13,22,49,52]. Among the agro-ecological areas, the average lamb birth weight is highest in the coastal region, followed by the Barind and Jamuna River Basin regions [13]. Higher birth weights were found in male lambs than in females [12,13,22]. However, lamb birth weight can vary depending on the season of birth, litter size (LS), lamb sex, nutritional status of the dam, management, and area [13,22,52–54]. Low birth weights and increased lamb mortality are caused by the dam’s poor nutritional status and health [52,53]. Though advanced research in the genetic and molecular fields has taken place in different countries, there is no such work on the growth performance or birth weight of lamb in Bangladesh.

**Table 2.** Morphometric features of native sheep.

Type of native sheep	Sex	Wither height (cm)	Heart girth (cm)	Body length (cm)	Ear length (cm)	Head length (cm)	Head width (cm)	Tail length (cm)	Scrotal circumference (cm)	Testis length (cm)	References
Jamuna River Basin	Male	46.80 ± 1.30	61.53 ± 1.23	43.33 ± 1.15					18.20 ± 0.51	10.67 ± 0.45	[10]
	Female	49.15 ± 0.31	57.88 ± 0.44	51.65 ± 0.46							
Jamuna River Basin	Male	52.10 ± 5.37	57.0 ± 6.32	50.61 ± 5.92	9.74 ± 2.25	18.63 ± 3.03	13.34 ± 3.10				[22]
	Female	49.15 ± 3.48	54.82 ± 4.48	49.26 ± 4.40	8.55 ± 3.39	18.19 ± 2.21	12.83 ± 2.09				
Barind sheep	Male	54.78 ± 1.88	67.80 ± 4.46	54.90 ± 3.13	7.13 ± 3.21	20.06 ± 1.28	12.15 ± 1.09				[21]
	Female	52.53 ± 2.52	70.41 ± 6.96	55.69 ± 3.06	7.30 ± 3.17	18.90 ± 1.45	11.36 ± .78				
Indigenous (Non-descriptive)	Male	58.47 ± 0.75	68.59 ± 0.74	58.88 ± 0.83	11.23 ± 0.32	16.15 ± 0.24		10.83 ± 0.23	16.33 ± 0.18		[49]
	Female	49.02 ± 0.28	59.77 ± 0.27	49.08 ± 0.30	8.63 ± 0.17	15.04 ± 0.12		10.55 ± 0.15			
Coastal		53.5	64.9	45.9	8.4	16.3	7.8	12.0		10.7	[13]
Jamuna River Basin		51.7	62.7	41.5	8.4	16.0	7.7	12.0		10.7	
Barind		52.9	61.5	43.4	3.2	16.1	7.4	11.5		11.0	
Coastal		56.30	67.50	67.60	11.40	25.20					[12]
Jamuna River Basin		47.71	58.57	64.42	8.28	20.85					
Barind		48.50	60.20	65.70	6.40	19.75					

### Body weight

Growth is the only outstanding important trait, a response indicator for sheep and other farm animals’ production [55]. The mature body weight of native ram is ranged from 14 to 25 kg, and in the case of ewe, it ranges from 14 to 20 gm, respectively [4,12,21,22,49,56,57]. Among the different types of native sheep according to the agro-ecological area, the Coastal sheep comprise the highest growth performance, followed by Jamuna River Basin and Barind [12,13,49]. Average daily weight gain is ranged from 44 to 92 gm/day, which can vary with the effect of location, type of sheep, sex, season, nutrition status and milk production of the dam, quality of feed, management system, and heat stress [4,8,12,58].

## Feeding and Nutrition

### Feeding behavior

Sheep do not have a particular feeding habit, and most importantly, they do not require a lot of feed. They can eat grass and leaves from the pasture themselves. Ouédraogo-Koné et al. [59] and Mysterud [60] stated that small ruminants such as sheep and goats have different feed consumption patterns in pasture areas; sheep are mainly grazers, while goats are more likely to browse. Sheep can consume different diets based on local plants, which allows these animals to adapt to new habitats under many environmental conditions, presenting greater flexibility in their behavior [61,62]. If they have a choice of feed resources, they will be selective in their feeding behavior. Khaskheli et al. [63] noted that goats spend more time eating than sheep while ruminating time remains longer in sheep than goats. Sheep, on the other hand, tolerate less bitter feed than goats and frequently browse plants rather than grass [64]. Sheep begin grazing by performing a visual assessment of the pasture, establishing references to the quality and quantity of available forage. If the forage height is below the established mean, the animal moves in search of a place that ensures good forage intake [65]. It can efficiently nibble tiny blades of grass with its small muzzles and split upper lips compared to other animals [24]. Sheep have a natural preference for grass and are thus grazers, obtaining natural or cultivated forage directly from the field. Sheep are more negatively affected by drought because herbaceous plants are more sensitive to periodic moisture stress than woody plants. Grazing ruminants, goats, and sheep are known to consume a particularly broad range of browse leaves and are reported to select those that meet their nutritional needs and avoid those that can be toxic [66]. 

### Feed intake and nutrient digestibility

Feed intake measures an animal’s appreciation, selection, and consumption of its diet [67]. It is considered one of the main factors determining the potential of animal performance. Sheep were usually grazed around homesteads, roadside pastures, and fallow land. They also feed on tree leaves like mango, jackfruit, and ipil ipil [68]. Sheep are allowed to graze during the day on natural pasture, homestead forest, and fallow land. Sheep were assigned randomly into two feeding systems: the traditional feeding system and the improved feeding system. Based on the traditional feeding regime, sheep are reared on fallow lands, roadsides, and canals [69]. In the improved feeding system group, each sheep consumed 250 gm of ready-to-feed concentrate feed in addition to grazing [70]. In Bangladesh, people mostly rear their sheep in traditional ways with little or no concentrate supplementation. Sheep production can be made more profitable by adding concentrate as a supplementary source of feed energy. Based on chemical composition, nutrient digestibility, and nutrient utilization efficiency, tree leaves prove to be excellent feed ingredients, especially for small ruminants, and provide potential feed for livestock during long dry seasons and wet seasons when insufficient plant biomass is available. Islam et al. [71] reported that 97.8% of the sheep farmers fed leaves and grasses to their sheep, and about 2.2% of the sheep farmers used concentrates as supplementary feed in addition to grass and leaves. Another study also found that 60% of the sheep grazed roadside grass, and only 33% grazed roadside and cultivated fodder during the rainy season [72]. Dry matter (DM) consumption was reported to be higher in sheep compared to goats under different available feeding regimes in Bangladesh. In contrast, under an intensive feeding system, crude protein (CP) consumption was found to be significantly higher in sheep, but neutral detergent fiber and acid detergent fiber (ADF) consumption was lower in sheep compared to goats [63]. Sultana et al. [58] stated that the digestibility of DM, organic matter (OM), CP, and ADF was low at the age of 6–9 months in castrated native Bengal lambs fed ad libitum urea molasses straw with a concentrated mixture of the rate of 1% of their body weight. Sultana et al. [73] reported a similar range of DM, OM, CP, and ADF digestibility and N-balance in the same age group of lambs fed different diets. Native lambs were fed pelleted total mixed rations (TMRs) instead of traditional loose concentrate with forage for fattening, and results showed that feed intake and average daily gain were higher when fed pelleted TMR, but nutrients apparent total tract digestibility and blood metabolites were not affected [74]. Similar findings were made by Islam et al. [75] on Garole sheep, who concluded that the complete feed system (TMR) is one of the latest developments in the feeding regime to make the best possible use of locally available feed resources.

### Pre and postnatal nutrition

Due to the high nutritional requirements for colostrum and milk production, parturition and lactation are considered the most critical and stressful periods of the ewe’s life cycle because energy is the primary requirement of animals. The energy used by the animal is derived primarily from energy intake and secondarily from the mobilization and metabolism of body reserves [76]. Lamb growth, weaning, and survival influence ewe nutrition during pregnancy and lactation [77,78]. The nutrients available to ewes during pregnancy positively affect both udder development and subsequent lactation performance [79]. Also, the dam`s ability to produce milk for its offspring is a key driver for neonatal growth and development [80]. Besides, colostrum production was significantly (*p *< 0.01) affected by nutritional manipulation, while the chemical composition of colostrum was not different except for total solids. Ewes can be supplemented with high energy or high protein for 2 months before parturition, which influences physiological states, increases lamb growth performance, decreases ewe loss, and increases colostrum production, which is beneficial to lamb survival [81]. Ahmed et al. [82] supplied different levels of pre- and post-natal diet to native Bengal ewes at 7 weeks of gestation under a semi-intensive system and found significantly higher daily milk yield (*p *< 0.01), weaning weight (*p *< 0.05) and daily weight gain of lamb until weaning (*p *< 0.05) in the group supplementing concentrate mixture at the rate of 1.5% of their body weight. To achieve the best performance from ewes and their lambs, the authors concluded that a concentrated diet containing 20% CP should be fed from late pregnancy through lactation. Several authors [52,83–87] found that inadequate nutrition during late pregnancy and lactation can reduce birth weight, mammary development, and milk yield [52,86]. Moreover, the plane of nutrition is very important for ewes during pregnancy and lactation, and feeding the ewes high-ME-content feed can improve growth and milk production performance without any negative effect on blood metabolites [88]. 

## Reproductive Performances

Reproductive traits are revealed as the most economically important trait in animal production. Moreover, high reproductive performance is a significant feature and a major component of improving the production proficiency of livestock [89]. As a result, animals with an elect reproductive profile are considered a critical genetic resource for their genetic development. In sheep reproduction, age at first lambing (AFL), gestation length (GL), LS, lambing interval (LI), service per conception rate (SPCR), and post-partum heat of the breed is considered the key reproductive traits.

### Age at first lambing (AFL)

AFL is one of the vital postpartum traits of animals when a breeding ewe gives birth to an offspring. The AFL of some indigenous sheep in diverse agro-ecological zones of Bangladesh is summarized in Table 3. There is no significant variation in AFL among them, and the average value is 423.32 ± 14.91 days. However, when the sheep of three agro-ecological zones were compared, Barind sheep had the lowest AFL values, and Jamuna river basin sheep had the highest [12,13,90]. However, in small ruminants, AFL is closely allied to growth rather than age, and it is an innate characteristic. Still, few variations arise due to the influence of different factors, like genotype or breed, feeding and nutrition, environmental issues (temperature, climatic condition, and season), better management, body weight gain, and the presence of a ram in the flock [89].

### Gestation length (GL)

In an ewe, the approximate GL is 147 days, and it is not affected by age, birth weight, body condition score, litter type, or lamb genotype [91]. However, in male progeny and single progeny (non-multiple births), a longer GL is observed [92]. In Bangladeshi native sheep, there is no significant variation in GL, and the average GL of indigenous sheep is 149.19 ± 1.25 ranging from 147 to 152 days. The highest GL is observed in the Jamuna river basin; in Barind and coastal regions, the value is almost similar. 

### Litter size (LS)

LS is mainly influenced by the ovulation rate and is the main determinant of reproductive proficiency. Ovulation rates differ between sheep breeds; they increase with ewe age and are higher in the first half of the breeding season for seasonally breeding ewes [93]. As a result, LS increases with dam age (up to the fourth parity) and then decreases slightly [94]. The average LS of Bangladeshi native sheep is 1.92, ranging from 1.82 to 1.99, and there is no significant difference in LS considering different types of sheep in Bangladesh. However, coastal sheep have the largest LS, and Barind sheep have the smallest litter. 

### Lambing interval (LI)

The number of days between consecutive parturitions is known as the LI, which is one of the major components of reproductive performance and has an important impact on a sheep production enterprise. LI is affected by the breed, season, parity, and post-partum weight of the dam [95]. The average LI of Bangladeshi sheep is 149 days. Furthermore, there is no significant difference between different types of Bangladeshi sheep, with the highest LI (195.87) found in the Jamuna river basin and the lowest LI (193.18) found in coastal sheep. 

### Service per conception rate (SPCR)

SPCR is usually considered to be the number of ewes exposed to the ram for successful lambing. The average SPCR of Bangladeshi sheep is about 1.29 (Table 3). Many factors, such as age, LI, breed, and production system, influence the SPCR of sheep. However, the SPCR in different types of indigenous sheep in the country is nearly identical. 

### Postpartum heat (PPH)

The PPH or postpartum interval is the period from parturition until the first postpartum estrus accompanied by ovulation. The length of the PPH varies among domestic animals. In the ewe, the PPH for most breeds extends from lambing in the spring until the resumption of estrous activity in the autumn. The average PPH among the indigenous sheep of Bangladesh is about 38.50 days, whereas the highest PPH (41.95) is observed on coastal sheep and the lowest (35.63) on Jamuna river basin sheep (Table 3). 

## Production Performances

### Lamb production

Native sheep are a non-popular species raised primarily for meat production in Bangladesh [8,54], but several necessary steps have recently been taken to boost sheep production [54,70]. Different research findings revealed that economic lamb production could be achieved by semi-intensive and intensive rearing systems with an optimum slaughter or market age ranging from 6 to 9 months [49,52,58,82,96]. All the available types of native lambs have the potential for commercial lamb production, especially coastal lambs, which grow faster in terms of daily weight gain [8]. With concentrate supplementation at 1.5% body weight of the ewe throughout late pregnancy [52,82]; straw-based complete pellet feed that comprises 40% roughage (rice straw) and 60% concentrate [74]; and Moringa leaf or Moring foliage as an alternative for conventional concentrate mixtures [69,73,97]. Because the cost-effectiveness of lamb production is strongly affected by nutritional management during the production cycle, breed, sex, LS, birth weight, body weight, age, season, location, flock size, and management system also influence the growth and production performances of lamb [4].

**Table 3.** Reproductive performances of different indigenous sheep in Bangladesh.

Reproductive traits	Type of indigenous sheep	Value (mean ± SE)	NAR	Study population	References
AFL	Jamuna river basin	438.17 ± 26.88	3	92	[10,12,13,49]
	Barind	405.97 ± 27.78	4	178	
	Coastal	429.53 ± 27.12	4	205	
	Average	423.32 ± 14.91	11	475	
GL	Jamuna river basin	152.13 ± 0.67	2	92	
	Barind	147.66 ± 2.66	2	178	
	Coastal	147.78 ± 1.78	2	205	
	Average	149.19 ± 1.25	6	475	
LS	Jamuna river basin	1.97 ± 0.22	3	92	
	Barind	1.82 ± 0.12	4	178	
	Coastal	1.99 ± 0.23	4	205	
	Average	1.92 ± 0 .10	11	475	
LI	Jamuna river basin	195.87 ± 13.00	3	92	
	Barind	194.18 ± 13.64	4	178	
	Coastal	193.18 ± 11.92	4	205	
	Average	194.27 ± 6.72	11	475	
SPCR	Jamuna river basin	1.31 ± 0.09	3	92	
	Barind	1.29 ± 0.09	4	178	
	Coastal	1.28 ± 0.08	4	205	
	Average	1.29 ± 0.04	11	475	
PPH	Jamuna river basin	35.63 ± 2.62	3	92	
	Barind	37.20 ± 5.02	4	178	
	Coastal	41.95 ± 1.73	4	205	
	Average	38.50 ± 2.04	11	475	

SE, standard error; NAR, number of articles that reported the parameters.

### Carcass characteristics

Among the available small ruminants, there is a clear difference in price and acceptance between sheep and goat meat, though both types are nearly similar in characteristics and quality [57]. More specifically, saturated fatty acids, monounsaturated fatty acids, polyunsaturated fatty acids, and some sensory characteristics, *viz.* Sheep meat has superior juiciness, tenderness, and other characteristics to goat meat [57,98]. The proximate composition of native sheep meat in different feeding systems is described in Table 4. In general, the slaughter age varied from 1 to 3 years with a live body weight of 8–22 kg with the traditional rearing system [57,99], but to gain economic benefit, the optimum slaughter age of native sheep is 6–9 months with a live body weight of 18.6–23.4 kg [58,82,100]. Sheep dressing percentages range from 39% to 68%, depending on age, breed, sex, feeding, and production system [57,58,82,99]. However, no significant difference is reported in meat quality and sensory characteristics between different production systems [101].

**Table 4.** Proximate composition of sheep meat.

Components	Dry matter (%)	CP (%)	Fat/ ether extract (%)	Minerals/ ash (%)	References
Organic system		22.41	3.6		[101]
Traditional system		23.84	4.4		
Roadside grass and concentrate feed		22.09	4.16	1.14	[75]
Pellet feed		21.19	4.16	1.14	
Native sheep	27.99	23.65	4.03	0.90	[57]

The major sheep population of Bangladesh is indigenous, and their wool is mainly coarse in nature. Approximately 2.5 thousand metric tons of raw wool are produced each year, with an average shearing rate of 440 gm per sheep and an average shearing rate of 800 gm per sheep per year [12,13,102,103], a fine wool percentage of around 17.07%, a hairy wool percentage of around 74.33%, and a staple length of 7.47 cm [13]. However, this potential by-product mostly goes to waste due to a lack of appropriate processing and applications [102,103]. Wool production also has a close association with the nutritional status of sheep [104,105]. Wool can be used in various finished products to meet daily needs in the modern era, thanks to the development of many locally made fibrous materials. In a joint research report, BLRI and Bangladesh Jute Research Institute reported that 30% wool, 30% jute, and 40% cotton fiber 12 sec blended yarn was economically more feasible for the development of blended yarn with jute, cotton, and wool, which may open a new window for the development of diversified handicraft products [102].

## Diseases and Mortality

Different health problems in sheep are the main threat to sheep farmers in Bangladesh regarding production loss. Infectious diseases (bacterial and viral) and endo-parasitism wreak havoc on sheep health, resulting in significant production losses and financial losses for farmers due to lower reproductive rates, lower feed conversion efficiency, higher production costs, increased risk of zoonotic diseases, and public health risks. The most common clinical illness with so-called diarrhea and pneumonia [13], along with Peste des Petits Ruminants (PPR), foot rot, and gastrointestinal infection, causes serious health hazards [106]. Different factors, including age, sex, and season, aggravate the lamb mortality rate of 12.4% in native sheep. In contrast, it is about 7.0% in July through October, 17.6% in November through February, and 12.5% in March through June, respectively [107].

Enterotoxaemia in sheep, also known as pulpy kidney disease or overeating disease, is mainly caused by *Clostridial perfringens* type D with high carbohydrate diets, which affects faster-growing lambs that exhibit uncoordinated movements and convulsions before death [108]. In Bangladesh, the prevalence of enterotoxaemia is around 0.07%, with a year-to-year prevalence of around 0.05% and a season-to-season incidence of around 0.05% [108–110].

Brucellosis, or Malta fever in sheep, is mainly caused by *Brucella* spp., are facultative intracellular parasites that may cause abortion in the early stages of gestation, orchitis in male animals along with infertility, and also decrease the milk yield of ewes [111].

Pneumonia causes the death of about 30% of feedlot cattle and high mortality in sheep worldwide [112]. *Pasteurella multocida* and *Pasteurella hemolytica*, mainly commensal organisms, are the main etiological agents for pneumonia in sheep. The incidence of pneumonia in native sheep of Bangladesh is about 1.02%; however, it is more prominent in the winter season (0.53%) than in the rainy season (0.37%) [110].

PPR is the most lethal and devastating viral disease of small ruminants, including native sheep. It is primarily an endemic disease in Bangladesh, causing high fever, diarrhea, dehydration, coughing, sneezing, necrotic stomatitis, and, to a lesser extent, pneumonia that leads to bronchopneumonia [113,114]^114^]. PPR in sheep has a prevalence of about 9.17%–18.18% reported in different areas of Bangladesh [114].

Contagious ecthyma is a common viral disease of small ruminants, characteristically caused by a parapoxvirus of the subfamily Chordopoxvirinae, also known as orf, sore mouth, or contagious pustular dermatitis [115]. Lesions occur around the lips, gums, eyelids, and oral cavity and are also found in teats and feet [116], along with internal organs like the esophagus, stomach, and intestine of the affected animals, which may be found by post-mortem examinations [117]. Regarding seasonal variations [111], young animals are more susceptible to this disease than adults.

A poxvirus causes sheep pox, another viral infection of a zoonotic nature that belongs to the family Poxviridae under the genus Capripoxvirus, in small ruminants [118]. It is a sheep disease that causes nodule formation in unwooled skin, high body temperature, and some keratitis [119]. However, the prevalence of sheep pox is rare; it mainly occurs in confined flocks in Bangladesh.

Nevertheless, parasitism, specifically gastro-intestinal parasitic infection, seriously hampers sheep production in Bangladesh, which mainly depends on age, sex, season, pasture, and geographical location [120]. Islam et al. [106] reported that sheep with a free-range grazing system were highly susceptible to helminth infections (82.8%) rather than those with a semi-intensive management system. Seasonal variation is also a drawback for sheep rearing; about 97.41% prevalence was found in the rainy season, about 91.31% in the summer, and in the winter, it was about 82.35%. They also found that young sheep (89.27%) were more susceptible to helminth infection than adults (81.11%) and kids (72.41%). Moreover, other internal parasites like roundworms and hookworms have the same devastating impact with profuse diarrhea, dysentery, anorexia, and anemia. Garole sheep have some characteristics of grazing in lowland areas rather than highlands, which gives them some degree of resistance against internal parasites, including liver fluke [121] and trematode infection [122]. On the other hand, external parasites like ticks, mites, and lice cause economic losses to farmers with wool-producing sheep, where lice infestation was found at about 58%, and psoroptic mange caused infection at about 12%, respectively [123].

Nonetheless, the most common metabolic diseases of sheep are hypocalcemia, hypomagnesemia, and pregnancy toxemia, which primarily affect the periparturient stage of their lifecycle. Nutritional scarcity in the last few days before parturition and during the first lactation may occur [124]. Hypocalcemia, or a lack of calcium, is most common in ewes a few weeks before lambing and can last for months. In ewes, hypomagnesemia can occur between 2 and 8 weeks of hypocalcemia, hypomagnesemia, and pregnancy toxemia, which primarily affect the periparturient stage of their lifecycle. Nutritional scarcity in the last few days before parturition and during the first lactation may occur [124]. Hypocalcemia, or a lack of calcium, is most common in ewes a few weeks before lambing and can last for months. In ewes, hypomagnesemia can occur between 2 and 8 weeks post-lambing, especially during the spring, when rapid-growing spring grasses are relatively low in magnesium, which is a must in the daily diet of sheep. Pregnancy toxemia is another metabolic illness characterized by low blood glucose and high ketone bodies in the last 6 weeks of pregnancy. Ewes carrying multiple fetuses face problems when nutritional intakes are limited by maximum energy demands [125].

## Prospects and Problems of Indigenous Sheep Farming in Bangladesh

There is a vast scope for sheep farming in developing countries like Bangladesh, which may create sustainable opportunities by generating income and finally contributing to the economy of the country. It is one of the most important small ruminants and has great potential to create a meat revolution in Bangladesh through profitable lamb production [4,22,126]. Moreover, by producing different diversified products, wool can contribute as a valuable by-product with economic benefits [13,127]. Indigenous sheep are an important part of rural communities’ socioeconomic structures [128], and they are regarded as “cash income” by rural farmers due to their easy availability for sale or exchange [129]. As they do not have a particular feeding habit, it is easy to maintain them in a small or medium space with naturally grown grasses, low-quality roughages, crop residues, tree toppings, farm and vegetable waste, aquatic weeds, and grasses in knee-deep water with a minimum concentrate mixture or any supplementation [49,71,100,106,130,131]^131^]. Sheep farming could become a sustainable way to produce animal protein, end poverty, give women more power, and help Bangladesh’s economy grow if farmers had more knowledge and training about available technologies, motivation, intensive management, and controlled breeding with superior rams [21,100,131,132]^132^].

Though sheep are a potential livestock species, there are some problems and limitations regarding sheep farming in Bangladesh. Because most sheep farmers are illiterate or undereducated, they cannot facilitate environmentally friendly flock management [133]. Sheep are less popular than goats in terms of cultural acceptance due to negative marketing messages about the flavor and quality of their meat [52,133]. Besides that, most farmers did not get loan facilities from any government or private organization as they were small-scale farmers or had inadequate assets to mortgage [126,133]. On the other hand, the genetic improvement of a species is largely dependent on breeding superior animals to produce the next generation. But less attention has been paid to improving the sheep at the field level, resulting in the loss of valuable germplasm. Only a few government institutes practice modern breeding and reproductive technologies, and they take some initiatives to improve native sheep [131,134]. Lack of sufficient breeding rams tends to result in crossbreeding or inbreeding of the native sheep, resulting in genetic erosion [135]. Another major constraint is a lack of feed, which is the result of a combination of factors such as a lack of pasture land, high feed costs, competition between human and animal feed, and high prices for vitamins, minerals, and other supplements [13,71,132,136]. Poor health management practices, along with inadequate knowledge and inadequate veterinary and diagnostic services, a lack of vaccine facilities, a high price for medicines, a lack of consciousness, and an effective prevention and control policy, cause economic losses in sheep farming in Bangladesh [8,110,132]. Furthermore, marketing strategies are another major obstacle to the smooth development of sheep production in Bangladesh, including a lack of market information, a lower price, and a lack of organized and structured markets for meat and wool [126,132].

## Conclusion

Sheep farming plays an important role in improving the livelihoods of small farmers in Bangladesh, significantly contributing to fulfilling the national demand for animal protein. However, the reproductive and productive performance of native sheep differs depending on their sex, birth type and weight, parity, management system, location, and year’s season. The scarcity of quality rams under field conditions, lack of an improved breed, the high price of feed, lack of medicine, and lack of credit and marketing facilities hamper profitable sheep farming. Sheep production communities need to be developed to reduce existing challenges, and government and nongovernment organizations should organize related training for farmers and entrepreneurs. Because adequate housing, nutritional modification, and health management can improve reproductive and productive performance while reducing environmental stress responses, To combat the multiple challenges of sheep farming in Bangladesh, more research efforts are needed in the fields of nutrition, reproduction, and health management to provide time-based assistance to the farmers.

## List of abbreviations

ADF, Acid detergent fiber; AFL, Age at first lambing; BLRI, Bangladesh Livestock Research Institute; CP, Crude Protein; DNA, Deoxyribonucleic Acid; DM, Dry Matter; GL, Gestation length; LS, Litter size; LI, Lambing interval; OM, Organic Matter; PPH, Postpartum heat; PPR, Peste des Petits Ruminants; SPCR, Service per Conception rate; TMR, Total mixed ration.
